# LOSITAN: A workbench to detect molecular adaptation based on a *F*_*st*_-outlier method

**DOI:** 10.1186/1471-2105-9-323

**Published:** 2008-07-28

**Authors:** Tiago Antao, Ana Lopes, Ricardo J Lopes, Albano Beja-Pereira, Gordon Luikart

**Affiliations:** 1 Liverpool School of Tropical Medicine, Pembroke Place, Liverpool L3 5QA, UK; 2 REQUIMTE, Departamento de Química, Faculdade de Ciências, Universidade do Porto, Rua do Campo Alegre, 687, 4169-007 Porto, Portugal; 3 CIBIO, Centro de Investigação em Biodiversidade e Recursos Genéticos, Campus Agrário de Vairão, Universidade do Porto, Portugal; 4 Division of Biological Sciences, University of Montana, Missoula, MT 59812, USA

## Abstract

**Background:**

Testing for selection is becoming one of the most important steps in the analysis of multilocus population genetics data sets. Existing applications are difficult to use, leaving many non-trivial, error-prone tasks to the user.

**Results:**

Here we present LOSITAN, a selection detection workbench based on a well evaluated *F*_*st*_-outlier detection method. LOSITAN greatly facilitates correct approximation of model parameters (e.g., genome-wide average, neutral *F*_*st*_), provides data import and export functions, iterative contour smoothing and generation of graphics in a easy to use graphical user interface. LOSITAN is able to use modern multi-core processor architectures by locally parallelizing fdist, reducing computation time by half in current dual core machines and with almost linear performance gains in machines with more cores.

**Conclusion:**

LOSITAN makes selection detection feasible to a much wider range of users, even for large population genomic datasets, by both providing an easy to use interface and essential functionality to complete the whole selection detection process.

## Background

Understanding the contribution of selection and molecular adaptation in shaping genome wide variation is among the most exciting and widely researched problems with many applications ranging from human health to conservation of endangered species. Among the many selection detection strategies [1], *F*_*st *_outlier approaches are becoming widely used [2,3] because they are important not only for studying the genetic basis of adaptation but also for eliminating non-neutral outlier loci from data sets before computing most population genetic parameters (e.g., *F*_*st*_, *N*_*m*_, *N*_*e*_), that require neutral loci [4]. This is particularly important in a time where production of data sets with information from hundreds of loci is becoming fairly common.

One such *F*_*st *_method is described in [2,5] (but see also [6] and [7]) and is implemented in the fdist program and can be used for any codominant genetic molecular markers including microsatellites, Single Nucleotide Polymorphisms (SNPs) and allozymes. This method evaluates the relationship between *F*_*st *_and *H*_*e *_(expected heterozygosity) in an island model [8], describing the expected distribution of Wright's inbreeding coefficient *F*_*st *_vs. *H*_*e *_under an island model of migration with neutral markers. This distribution is used to identify outlier loci that have excessively high or low *F*_*st *_compared to neutral expectations. Such outlier loci are candidates for being subject to selection.

Using fdist can be a challenging task for those not familiarized with command-line applications and requires a specific data format not used by other applications [9]. Furthermore, several independent runs are usually needed to tune parameters (e.g., determine the appropriate average *F*_*st*_) before a final execution is made in a process that is prone to human introduced mistakes. Fdist, not being one of the most computationally intensive programs available, can still take up to one hour for a single run (especially if smooth contours for confidence intervals are required), and, in most cases, multiple runs are needed for parameter tuning. Large population genomic datasets can take even longer. In this context, fdist requires experienced computer users, and its usage is error prone (e.g., by incorrectly converting data files or not approximating average *F*_*st *_appropriately).

## Implementation

**Figure 1:**
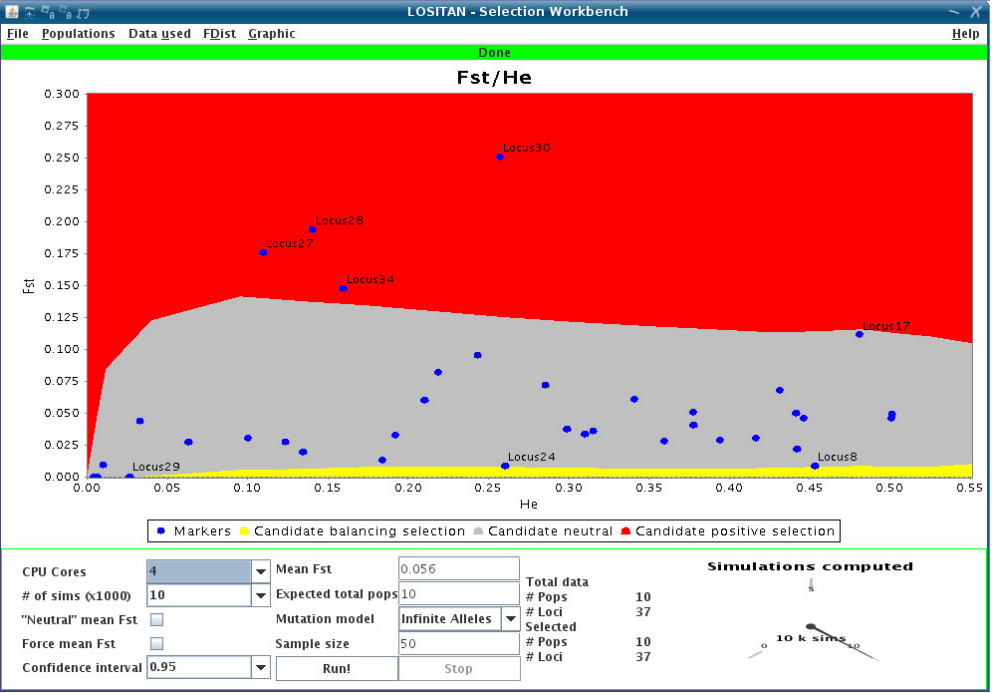
**LOSITAN console**. Screen shot showing run parameters (bottom panel) and a graphical output with the simulated confidence area for neutral loci (middle color band) with loci from the original empirical dataset represented as dots. Outliers are tagged with labels.

We designed LOSITAN (LOoking for Selection In a TANgled dataset), a selection detection workbench constructed around fdist. LOSITAN is a Java Web Start application coded mostly in Jython with a small part in Java, allowing direct execution from the web. LOSITAN provides the following features:

1. Easy to use interface (Figure 1), directly usable from the web.

2. Data import in Genepop [10] format.

3. Generation of graphics in several formats (PNG, SVG and PDF).

**Figure 2:**
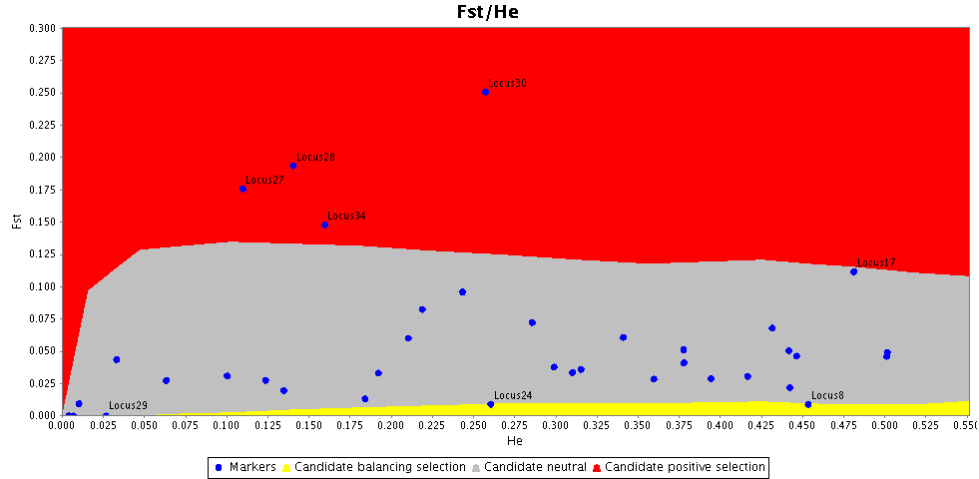
**PNG Output**. Graphical output from LOSITAN in PNG format without any post-edition.

Graphics can be generated in several formats (covering both bitmap and vector format styles) and parametrized in many ways (from choosing colours to deciding which labels are printed, among others). A completely unedited example of a PNG output is presented on Figure 2.

4. Data export in a format suitable for import into statistical packages like R [11] or commonly used spreadsheet software.

In case the user desires to further analyze the data or have total flexibility in generating graphics, LOSITAN makes available both the confidence intervals computed and the *F*_*st*_s and heterozygosities for each locus.

A simple R script is supplied in order to facilitate loading the data into R. Loading in spreadsheet software is done simply by importing as a tab delimited file.

5. Choice of which populations and/or loci are studied.

6. Approximating mean neutral *F*_*st *_(in the real dataset) by removing potential selected loci.

The initial mean dataset *F*_*st *_is often not neutral in the sense that (initially unknown) selected loci are often included in the computation. LOSITAN can optionally be run once to determine a first candidate subset of selected loci in order to remove them from the computation of the neutral *F*_*st*_. This value will be, in most cases, a better approximation of the neutral *F*_*st*_[5]. The procedure works as follows: LOSITAN is run a first time, using all loci to estimate the mean neutral *F*_*st*_. After the first run, all loci that are outside the desired confidence intervals (e.g. 99% CIs) are removed and the mean neutral *F*_*st *_is computed again using only putative neutral loci that were not removed. A second and final run of LOSITAN, using all loci, is then conducted using the last computed mean. This procedure lowers the bias on the estimation of the mean neutral *F*_*st *_by removing the most extreme loci from the estimation. Naturally all loci will be present in the last run will have their estimated selection status reported.

7. Approximating average simulated *F*_*st *_to the average value found in the real dataset even when the experimental conditions are far from the ones where the theoretical formula $F_{st}=\frac{1}{4Nm+1}$ holds (e.g. low number of demes or the usage of the stepwise mutation model, common in microsatellite markers).

To be able to (optionally) approximate the average *F*_*st *_in conditions far from the theoretical optimum, LOSITAN starts by running fdist for 10,000 realizations using the theoretical value, calculating the average simulated *F*_*st*_, if the value is too far from the real average *F*_*st*_, LOSITAN uses a bisection approximation algorithm running 10,000 realizations for every tentative bisection point. The algorithm works by iteratively slicing the interval of possible *F*_*st *_values (i.e., between 0 and 1) in half at each iteration and choosing the mean of the bounds on each iteration (with the exception of the first iteration where one of the extremes is chosen). An example is provided to make the approach clearer:

In a certain demographic scenario we want to simulate a neutral *F*_*st *_of 0.08. The algorithm starts by trying 0.08. If the result is higher than desired then 0.0 will be tried (creating an absolute lower bound limit), after that 0.04 (0.0 + 0.08)/2 will be tried, if the result is too low, 0.06 will be used next (i.e. (0.04 + 0.08)/2), the process repeats until the error margin is acceptable.

In practical terms the method was able to converge to the desired value in all cases tested (a completely trivial bisection approach is not possible as the method for computing *F*_*st *_is stochastic and results might vary for the same input conditions).

8. Iterative smoothing of confidence interval contours.

Contour smoothing is achieved by running fdist an extra 5,000 realizations. The user can request smoothing an unlimited number of times until the result is deemed satisfactory.

9. Ability to use multiple CPU cores and processors when running fdist.

To be able to use multiple cores, LOSITAN divides the number of desired simulation repeats among all available cores (although the application detects the number of existing cores, the user is able to change the number of simultaneous concurrent processes), this is possible because fdist simulation runs are independent, thus making parallelization a simple task. Tests show a near linear relationship between the number of cores used and performance gains, an existing 5–10% penalty is due mainly to joining the partial results together. LOSITAN, although being directly executable from the web is a client-side application and all computational intensive operations occur on the user computer and not on the server.

10. Automatic and transparent download of the latest version of fdist.

We maintain the latest version of the fdist application on the server, which is downloaded transparently by the client application whenever there is a new version. At the time of this writing the supported version is fdist2.

The interface includes tips for all the less obvious parameters and enforces constraints for all the user inputs which the system can infer are not correct.

## Results and discussion

In a beta test release to users the feedback was generally very positive stressing essentially that the application is easy to use, allows to easily input and output data and deal with non-trivial parameter determination like calculating neutral *F*_*st*_. Most importantly it made users aware of issues in data analysis that they were not aware of. For example, users were not aware of how to estimate the genome wide average neutral *F*_*st *_from their empirical data set by removing one or a few strong outlier loci, and the recomputing the average *F*_*st*_. Although LOSITAN helps avoid many pitfalls involved with using *F*_*st*_-outlier approaches in general, it is not able to solve fundamental issues regarding these approaches, for instance the non-linear behavior of $F_{st}=\frac{1}{4Nm+1}$ when *F*_*st *_approaches zero can make it difficult to detect low *F*_*st*_-outliers especially when selection is not strong. As such an easy to use application should not be seen by users as a excuse to avoid critical reasoning around the the whole selection detection process. Feedback from users also allows to chart possible future work, like supporting dominant markers or supporting other selection detection approaches like [3].

Our solution to use all the available computing power on new multi-core hardware is an example of an "embarrassingly simple parallel" computation approach. We contend that having a simple approach is a good principle: The point in this application is to make all computational power available to the users and not to develop new concurrent algorithms. A simple, highly efficient, elegant and less bug-prone approach is what responds to the users needs, as the objective of this work is not to develop new algorithms, but to use them.

## Conclusion

LOSITAN is built along the principles exposed in [12], namely that intuitiveness and user empowerment should be fundamental guidelines for software construction targeting biologists. This is done, not only by supplying an easy to use web interface for an, otherwise, hard to use application, but also allowing the use of widely utilized population genetic data formats, automating the tuning of nuisance parameters and lowering the computational costs on modern hardware. In addition, strong emphasis is put on trying to avoid errors on the usage of the software either by both enforcing constraints and giving suggestions on less obvious features. This will lower the barriers to usage of the underlying application, allowing for a wider user base which will be able to concentrate more on the biological problems and less on unnecessary application complexity.

We are in the dawn of the era of multi-core computing. The vast majority of existing software cannot make use of the extra computational power made available on new machines. Our approach, based on partitioning a computational intensive task into smaller ones, can be used to leverage the extra computational power even without changing existing code on applications which can be broken into smaller independent running units. This partitioning approach can be performed in some cases by users on existing software or by programmers in new applications that take advantage of multiple cores. With the current trend of supplying many more cores with new computers, strategies like the one presented here will be mandatory in order to take full advantage of all the existing processing power. LOSITAN is one of the first of many applications to explore the multi-core programming paradigm.

Future planned developments will include addition of other F-outlier methods and simulation facilities for explore the effects of different demographic scenarios on *F*_*st *_variance and the detection of outliers. All the code to handle GenePop and fdist file formats and applications was also donated to the Biopython project and is publicly available starting from version 1.44.

## Availability and requirements

**Project name **LOSITAN

**Project home page **. Development site: 

**Operating systems **Platform independent

**Programming language **Java and Jython

**Other requirements **Browser with JavaWebStart to run over the internet (software can be run locally).

Windows: At least Windows 2000 and Java 1.6.

Mac OS X: 10.4 (Tiger) and Java 1.5 (Most current 10.4 installations will require a freely available Java update).

Linux: Java 1.6 and the free GNU C compiler.

**License **GNU GPL

**Any restrictions to use by non-academics **None

## Authors' contributions

TA is the leading architect and main developer of LOSITAN, and drafted this publication. AB–P and GL have both theoretically drafted the idea of developing LOSITAN and together with TA, RJL contributed in discussions, planning and writing of this manuscript. RJL developed the web page and tutorials and AL developed the code regarding multi core detection and graphics and data export.
